# Effects of Land Use on the Soil Microbial Community in the Songnen Grassland of Northeast China

**DOI:** 10.3389/fmicb.2022.865184

**Published:** 2022-07-08

**Authors:** Guofu Liu, Zhenjian Bai, Guowen Cui, Wenhua He, Zelai Kongling, Guoxu Ji, Hao Gong, Dandan Li

**Affiliations:** ^1^Department of Animal Science and Technology, Northeast Agricultural University, Harbin, China; ^2^Qiqihar Grassland Station, Qiqihar, China

**Keywords:** land use type, bacterial community, fungal community, soil properties, plant community composition

## Abstract

Land use change obviously changes the plant community composition and soil properties of grasslands and thus affects multiple functions and services of grassland ecosystems. However, the response mechanisms of soil microorganisms, key drivers of the nutrient cycle and other soil functions during changes in grassland use type and associated vegetation are not well understood. In this study, Illumina high-throughput sequencing was used to analyze the changes in the soil microbial community structure of four grassland use types: exclosure (EL), mowed land (ML), grazed land (GL), and farmland (FL) in the Songnen Plain of Northeast China. The results showed that the FL and EL had significantly higher soil total nitrogen (TN) and lower soil electrical conductivity (EC) and pH than GL and ML. In contrast, the GL and ML had higher soil bulk density (BD) and organic matter, respectively, than the other land use types. In addition, the values of the Shannon diversity and Pielou’s evenness indexes were highest in the EL of all the land use types. Based on the high-throughput sequencing results, we observed high levels of α diversity in the FL for both bacteria and fungi. A structural equation model (SEM) revealed that pH and EC had a direct and positive effect on the bacterial community structure and composition. In addition, plant taxonomic diversity (according to the Shannon diversity and Pielou’s evenness indexes) indirectly affected the bacterial community composition via soil pH and EC. Notably, fungal composition was directly and positively correlated with soil nutrients and the value of Pielou’s evenness index changed with land use type. In conclusion, soil properties and/or plant diversity might drive the changes in the soil microbial community structure and composition in different grassland use types.

## Introduction

Grassland ecosystems are one of the most widely distributed terrestrial ecosystems in the world and cover more than 25% of the Earth’s surface (Wu et al., 2021). Grasslands perform many functions and ecosystem services, including creating a high-yield and high-quality forage supply, storing carbon, purifying water, controlling soil erosion, and maintaining biodiversity (Fuchslueger et al., 2019; Zhang et al., 2021). Many natural and human ecological processes, such as vegetation succession and changes in agricultural management measures, influence changes in grassland ecosystems. The most profound and directive factor is the change in land use. The impact on ecosystem function depends mainly on the type, severity, frequency, and timing of disturbances caused by land use (Wang X. Y. et al., 2020). These factors are related to the participation of soil microorganisms, which respond to changing soil conditions (Fang et al., 2020).

Land use change is currently one of the most important environmental changes and can alter soil environmental factors, nutrient conditions, and biological interactions, thereby affecting microbial communities (Engelhardt et al., 2018; Wang et al., 2019). Previous studies have reported that the conversion of woodland to pasture or farmland could lead to a reduction in the abundance and diversity of some microbial communities; this shift may be related to nutrient cycling because soil microorganisms and their activities play an important role in soil formation, organic matter decomposition, nitrogen fixation and other soil processes (Merloti et al., 2019; Shrestha et al., 2019). Similarly, the bacterial community in cultivated soil under intensive management was different from that in unmanaged soil (Drenovsky et al., 2010). More specifically, management measures, such as fertilization and herbicides, lead to more diverse soil microorganisms with higher functional redundancy, which is an important microbial strategy to overcome environmental interference (Szoboszlay et al., 2017; Santos et al., 2020). Furthermore, changes in plant community composition could alter the input of carbon resources to the soil by producing a variety of litter and root exudates, thereby indirectly changing microbial diversity and activity (Zhong et al., 2020). For example, fungal communities use carbon less efficiently and form less biomass than bacteria (Martí-Roura et al., 2019). However, the microbial communities that perform or participate in these processes in different land use types and their response characteristics to land use change still need to be determined and characterized.

Grazing, mowing, exclosure, and farmland are the main land use types in grasslands (Wang X. Y. et al., 2020). Grazing is a selective disturbance that can reduce the abundance of palatable plants and is one of the main grassland utilization strategies (Chen et al., 2021a). Moderate grazing contributes to improving plant diversity and productivity and may shape soil microbial communities with multiple functions. However, overgrazing reduces the structural and compositional complexity of plant communities and reduces litter and root exudates into the soil, thus affecting the availability of soil substrates (Dassen et al., 2017; Han et al., 2018). Mowing is a nonselective disturbance that changes the material cycle and energy flow of the entire grassland ecosystem, although it does not always produce the expected results (Chen et al., 2021b). Mowing can alter plant species diversity and improve plant community stability (Li et al., 2017). In addition, mowing can change the hydrothermal conditions by removing aboveground biomass, which can have positive or negative effects on soil properties and microbial communities (Yang et al., 2012). Exclosure refers to grassland previously used for grazing and mowing but has been completely conserved with no continuous activities, thus serving as a means of restoring degraded grassland through natural regeneration in northern China (Yan and Lu, 2015). Exclosures can be established to improve soil quality and produce positive feedback to the plant community, i.e., increased plant productivity. In addition, higher plant biomass provides a rich carbon source for soil, which stimulates microbial activities and accelerates nutrient cycling (Szoboszlay et al., 2017). Human population growth causes an increase in food demand and the conversion of more grassland to farmland, resulting in soil degradation. Specifically, these factors lead to a large loss of soil organic matter (SOM), which reduces the stability of soil aggregates, reduces soil fertility, weakens soil structure, and accelerates soil erosion (Li et al., 2021). However, the effects of different grassland use types on microbial community structure remain poorly defined.

The Songnen grassland, the largest meadow grassland in China, is located on the eastern edge of the Eurasian steppe belt and plays an important role in the feedback and regulation of nutrient cycling. Therefore, it is necessary to study the response mechanisms and key microbial processes of the soil microbial community in the Songnen Plain to four grassland use types (exclosure, mowed land, grazed land, and farmland) to provide a theoretical basis for the sustainable management of grassland ecosystems. The objectives of our study were to (1) evaluate the changes in the soil microbial community under different land use types and (2) test the relative importance of environmental factors driving the changes in soil microbial communities in four land use types.

## Materials and Methods

### Study Sites

This study area was in Qiqihar, Heilongjiang Province, China (123°55′12″E, 47°21′15″N; 140 m a.s.l.) which has a semiarid continental monsoon climate and is located in the mid-temperate zone. The mean annual precipitation is 466 mm, and 80% of this precipitation falls between June and August. The annual average temperature is 3.2°C, with a mean monthly temperature ranging from −25.7°C in January to 22.8°C in July. The soil type of the study area is classified as carbonate meadow soil according to the Chinese Soil Taxonomic Classification (Gong et al., 2011).

### Experimental Design

In 2019, we selected four sites of land use in the meadow steppe: (1) The exclosure (EL) covered 50 ha and was fenced in 2016, with no subsequent grazing or mowing. (2) The mowed land (ML) covered 140 ha and was enclosed using iron mesh fence around the EL; mowing to 6 cm occurred every year since 2005 in mid-August to make hay. (3) The grazed land (GL) was located around the EL and ML and was grazed once every calendar month from May to September with 6 sheep/ha, ending when the grassland residual height reached approximately 6 cm. (4) Farmland (FL), which has been cultivated for over 20 years, is also part of this grassland. The main crop is maize, which is sown in May and harvested in October. Crop productivity mainly depends on natural rainfall and materials are not returned to the field after harvest. In the FL, the farmers regularly removed non-target weeds and the composition was monospecific, thus not further investigated here. We selected a large single monitoring area, representative of each land use, in which the altitude, soil texture, and topography (slope direction and gradient) are similar, and all areas use the pseudo-replication procedure (Gilliam et al., 2018; Merloti et al., 2019). The detailed characteristics of the land use history of the study sites are shown in Table 1.

**TABLE 1:** Site descriptions of the different land use types.

Site	Location	Elevation (m a.s.l.)	Soil type (Chinese Soil Taxonomic Classification)	Management	Dominant vegetation/Crop
Exclosure (EL)	124°9′25″E, 47°17′51″N	152	Meadow soil	No anthropogenic interference	*Leymus chinensis* (Trin.) T., *Calamagrostis epigeios* (L.) Roth, *Triglochin palustris* L., *Hordeum brevisubulatum* (Trin.) Link, *Puccinellia tenuiflora* (Turcz.) Scribn. et Merr., *Potentilla anserina* L., and *Saussurea japonica* (Thunb.) DC.
Mowed land (ML)	124°19′18″E, 47°16′21″N	148	Meadow soil	Mowing was performed every year at the time of peak plant biomass (mid-August), leaving stubble height of approximately 6 cm	*Leymus chinensis* (Trin.) T., *Phragmites australis* (Cav.) Trin. ex Steud., *Puccinellia tenuiflora* (Turcz.) Scribn. et Merr., *Hordeum brevisubulatum* (Trin.) Link, *Polygonum sibiricum* Laxm., and *Inula japonica* Thunb.
Grazed land (GL)	124°13′52″E, 47°18′1″N	150	Meadow soil	Free grazing with 6 sheep/ha from May to September	*Artemisia scoparia* Waldst. et Kit., *Puccinellia tenuiflora* (Turcz.) Scribn. et Merr., *Suaeda glauca* (Bunge) Bunge, *Leymus chinensis* (Trin.) T., and *Echinochloa crus-galli* (L.) P. Beauv.
Farmland (FL)	124°13′49″E, 47°19′22″N	154	Meadow soil	Application of N:P_2_O_5_:K_2_O (17:18:10) every year, conventional tillage, and spring herbicide application	*Zea mays* L.

### Plant Sampling and Analysis

In mid-August 2019, three non-continuous repeat plots (150 m × 150 m) were established in each site, with a minimum distance of 0.2 km between the plots. Within each plot three 1 m × 1 m randomly selected subplots were used to estimate plant species richness based on the number of species and assess the relative cover of each species by vertically projecting canopy cover (%) for each species. In addition, the individual plant height was recorded in each subplot. Subsequently, the aboveground parts of the plants were clipped, leaving stems with a height of 6 cm from the ground, dried at 65°C for 48 h and weighed. Plant diversity indexes (including Shannon, Simpson’s, and Pielou’s indexes) were calculated according to Tscherko et al. (2004).

### Soil Sampling and Analysis

After removing the plant material in the nine subplots mentioned above, 3 individual soil cores within a soil depth of 0–20 cm were randomly collected using a soil corer (4 cm in diameter) from each subplot and manually mixed to form one sample. Then, three composite samples from subplots were mixed as samples of each plot. Finally, a total of 12 samples (4 sites × 3 reps) were collected for this study. The soil was placed in an individual sterile plastic ziplock bag, which was placed inside an ice-cooled container and transported to the laboratory immediately. The samples were sieved (2 mm mesh) to remove plants, roots, and gravel and then partitioned into two subsamples: one was air-dried for physicochemical analysis and the other was stored at −80°C for DNA extraction.

Soil samples (0–20 cm) were collected into aluminum boxes using cutting rings in each plot and dried at 105°C for 24 h to determine the soil bulk density (BD) (Deng et al., 2014). The soil organic matter (SOM) content was determined using the dichromate oxidation method in the presence of H_2_SO_4_ and titration with ferrous ammonium sulfate (Sparks et al., 1996). Soil pH and electrical conductivity (EC; important indicators of salinity) were determined with a glass electrode (PHSJ-4A, Shanghai, China) and conductivity meter (DDSJ-308F, Shanghai, China) after shaking the soil (air-dried)/water (1:5, w/v) suspension for 30 min (Bao, 2008). The total nitrogen content (TN) and alkali-hydrolyzable nitrogen (AN) were measured by classical Kjeldahl digestion and distillation azotometry after extraction with 0.02 M sulfuric acid (Hanon K9860, Shanghai, China) (Bremner and Mulvaney, 1982) and using the alkaline hydrolysis diffusion method (Lu, 1999), respectively. Available phosphorus (AP) was extracted using 0.5 M sodium bicarbonate (NaHCO_3_) (pH 8.5) and then determined using the molybdenum blue method (UV-752 Shanghai, China) (Bao, 2008).

### Soil DNA Extraction

For each soil sample, the total DNA was extracted from 0.5 g of fresh soil samples (−80°C) using HiPure Soil DNA Kit (Magen, Guangzhou, China) according to the manufacturer’s protocols. DNA concentration and purity were checked by the absorbance ratios at A260/A280 and A260/A230 using a NanoDrop™ 2000 spectrophotometer (Thermo Fisher Scientific, Wilmington, MA, United States). Amplification of bacterial 16S rRNA was performed for the V3-V4 region, and the primers were 341F (5′-CCTACGGGNGGCWGCAG-3′) and 806R (5′-GGACTACHVGGGTATCTAAT-3′). For the fungal ITS2 region, the primers ITS3_KYO2 (5′-GATGAAGAACGYAGYRAA-3′) and ITS4R (5′-TCCTCCGCTTATTGATATGC-3′) were used for amplification. PCRs were performed in triplicate in a 50 μL mixture containing 5 μL of 10 × KOD Buffer (New England Biolabs, United States), 5 μL dNTPs (2 mM), 3 μL MgSO4 (25 mM), 1.5 μL of each primer (10 μM), 1 μL of KOD polymerase, and 100 ng of template DNA. Amplification was performed as follows: 94°C for 2 min; 30 cycles at 98°C for 10 s, 62°C for 30 s, and 68°C for 30 s; and a final extension at 68°C for 5 min. The PCR products were purified using the AxyPrep DNA Gel Extraction Kit (Axygen Biosciences, Union City, CA, United States) according to the manufacturer’s instructions and quantified using the ABI StepOnePlus Real-Time PCR System (Life Technologies, Foster City, CA, United States). The libraries were sequenced with the Illumina HiSeq 2500 system (Illumina, United States) at Gene Denovo Biotechnology Co., Ltd. (Guangzhou, China).

### Sequencing Data Analysis

The fungal and bacterial sequences were analyzed using the Quantitative Insights into Microbial Ecology (QIIME) pipeline (version 1.9.1) (Caporaso et al., 2010). We assembled paired-end clean reads that were merged as raw tags using FLASH (Magoc and Salzberg, 2011), and the sequences with lengths less than 150 bp and/or quality scores lower than 20 were removed for further analysis. The average lengths of sequences for bacteria and fungi were 457 and 368 bp, respectively. To detect and remove chimeric sequences, the tags were compared to the reference database using the UCHIME algorithm (Edgar et al., 2011) and clustered into operational taxonomic units (OTUs) based on a threshold of ≥97% sequence similarity using the UPARSE (version 9.2.64) pipeline. Chimeras and non-target classifications (e.g., chloroplast, mitochondria, archaea) were checked and eliminated during clustering. The taxonomic identities of the bacteria and fungi were determined using RDP software (version 2.2) (Wang et al., 2007) based on comparison with the SILVA database (version 132) (Quast et al., 2013) and UNITE database (version 8.0), respectively.

### Statistical Analysis

To reduce the effects of sequencing depth on the downstream analyses, OTU abundance tables were rarefied to the minimum sequence numbers per soil sample (92,000 and 87,000 for bacteria and fungi, respectively), and soil microbial diversity (including the Chao1, Simpson, and Shannon indexes) were calculated in QIIME. One-way ANOVA with Tukey’s HSD test was used to analyze the differences in the biotic and abiotic properties, including plant alpha-diversity, soil alpha-diversity, and relative abundances of dominant phyla across plots. Spearman’s rank correlation coefficients were calculated to determine the relationships of microbial alpha diversity and relative abundances of individual taxa with soil and plant properties. Statistics were performed using SPSS version 19.0 statistical software (SPSS Inc., Chicago, IL, United States). Statistical significance was evaluated at *P* < 0.05. The results are expressed as the mean values and standard errors (SEs).

We used principal coordinates analysis (PCoA) based on Bray–Curtis distances to analyze the dissimilarity of plant communities and soil microbial composition at the species and OTU levels, respectively. Differences in the microbial communities between land management types were further tested by permutational multivariate analysis of variance (PERMANOVA) (Anderson, 2001) and multivariate homogeneity of groups dispersions (BETADISPER) (Anderson et al., 2006) using the “vegan” package in R (version 2.5.3). Bacterial and fungal taxa with significant differences between sampling groups were identified using the linear discriminant analysis (LDA) effect size (LEfSe) method, and an effect size threshold of 2 was used to identify biomarkers (Segata et al., 2011). LEfSe software (version 1.0) was used to identify organisms that were significantly different using a nonparametric Kruskal–Wallis rank sum test (Krustal and Wallis, 1952). In addition, we performed a redundancy analysis (RDA) using CANOCO 5.0 software (Dang et al., 2010) based on the abundances of OTUs to elucidate the associations among the soil properties, plant communities and microbial groups. Manual forward selection and a Monte Carlo test with 499 permutations were implemented to determine the significance of soil and plant properties on the microbial communities.

A structural equation model (SEM) was created using SPSS Amos 26 (AMOS IBM, United States) to evaluate the direct and indirect effects of land use types on bacterial and fungal communities. To prevent collinearity between variables, we divided the soil properties into two groups: (I) soil nutrients (including SOM and TN) and (II) soil pH and EC. All the indicators were dimensionally reduced by principal component analysis (PCA), and the first principal component (PC1) was extracted to represent each variable group. The PCOA axis 1 scores were used as indicators of bacterial and fungal community composition. The model was fitted by the maximum likelihood estimation method using the chi square test (χ^2^, *P* > 0.05), goodness-of-fit index (GFI > 0.9), Akaike information criterion (AIC) and approximate root mean square error (RMSEA < 0.05) to test the goodness of fit of the model (Guo et al., 2021).

## Results

### Effects of Land Use Types on Soil Properties and Plant Community Parameters

Soil pH, EC, and BD differed significantly among the land use types, with higher pH, EC, and BD recorded in the GL plots than in the ML, EL, and FL plots. The SOM content was highest in the ML and lowest in the GL and differed significantly among all the land use types. Compared with the other land use types, the highest soil TN content was observed in the EL plot (*P* < 0.05). A significantly higher soil AP content was observed in the FL (11.23 mg kg^–1^) than in the EL; however, the AP content was similar in the rest of the plots. The soil AN concentration varied from 50 to 107 mg kg^–1^ and was highest in the ML and EL (Table 2).

**TABLE 2:** Effects of different land use types on soil and plant properties.

Site	pH	EC (μS cm^–1^)	SOM (g kg^–1^)	TN (g kg^–1^)	AN (mg kg^–1^)	AP (mg kg^–1^)	BD (g cm^–3^)	AB (g m^–2^)
EL	8.73 ± 0.22c	209.43 ± 46.39c	22.49 ± 0.50b	1.25 ± 0.04a	93.33 ± 10.69a	3.61 ± 0.84b	1.40 ± 0.02d	231.28 ± 21.23a
ML	9.45 ± 0.22b	439.67 ± 104.77b	31.41 ± 2.00a	0.66 ± 0.09c	106.17 ± 14.57a	7.08 ± 1.11ab	1.48 ± 0.05c	222.74 ± 34.22a
GL	10.11 ± 0.03a	646.67 ± 100.13a	11.74 ± 1.43d	0.68 ± 0.11c	50.17 ± 10.69b	6.24 ± 0.67ab	1.72 ± 0.04a	97.19 ± 11.60b
FL	8.12 ± 0.17d	109.93 ± 8.59c	15.91 ± 2.13c	0.92 ± 0.04b	67.67 ± 14.57b	11.23 ± 5.39a	1.64 ± 0.03b	/

*EL, exclosure; ML, mowed land; GL, grazed land; FL, farmland; AN, alkali-hydrolyzable nitrogen; AP, available phosphorus; SOM, soil organic matter; EC, electrical conductivity; TN, total nitrogen; BD, bulk density; AB, aboveground biomass.*

*Values are the means ± SEs (n = 3).*

*Different letters represent significant differences between the means (P < 0.05).*

**FIGURE 1:**
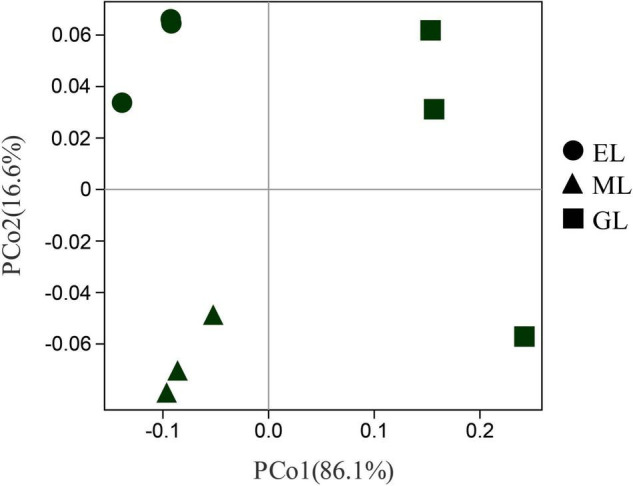
Principal component analysis (PCoA) of plant communities in different land use types. EL, exclosure; ML, mowed land; and GL, grazed land.

**FIGURE 2:**
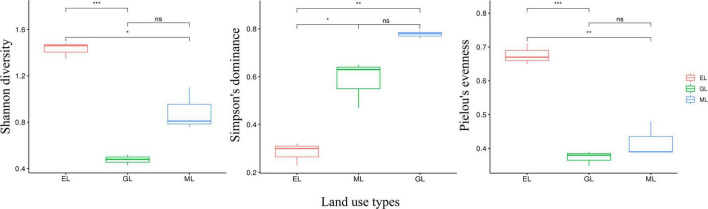
Plant alpha diversity in different land use types. EL, exclosure; ML, mowed land; and GL, grazed land. In the FL, the plant community composition was monospecific, thus it was not included in the analysis. *P < 0.05, **P < 0.01 and ***P < 0.001.

Principal coordinates analysis showed that the plant communities in the three land use types were significantly different (Figure 1; *P* < 0.05), and the plant Shannon diversity and Pielou’s evenness indexes in the EL were higher than those in the GL and ML, whereas Simpson’s dominance index showed the opposite trend (Figure 2). In addition, aboveground biomass was significantly lower in the GL than in the EL and ML (Table 2; *P* < 0.05).

### Effects of Land Use Types on Soil Microbial Community Diversity and Composition

A total of 1,223,170 and 1,176,336 high-quality sequences of bacterial and fungal communities were obtained from 12 samples after quality filtering steps. Bacterial sequences ranged from 92,489 to 107,410 per sample (mean = 101,931), whereas fungal sequences ranged from 87,512 to 106,646 per sample (mean = 98,028). The sequences were clustered into 32,269 bacterial and 6,267 fungal OTUs, with a 97% identity threshold. The Good’s coverage index for all plots was higher than 99.18 ± 0.68%, indicating that the sequencing effort was sufficient. For the bacterial community, the EL and FL had significantly higher Chao1 richness and Shannon indexes than the GL and ML, and no differences were found in the Simpson index among the plots. In the fungal community, a significantly higher Shannon index was observed for the FL than for the EL, while there were no significant differences among plots for the Chao1 and Simpson indexes (Table 3). Spearman’s analysis showed that soil pH, EC, and the plant Simpson index were significantly negatively correlated with bacterial richness according to the Chao1 index (*r* = −0.967, *P* = 0.033; *r* = −0.955, *P* = 0.045; *r* = −0.979, *P* = 0.021, respectively), while the fungal Shannon diversity was positively correlated with AP (*r* = 0.977, *P* = 0.023) and negatively correlated with the Shannon and Pielou’s indexes for the plants (*r* = −0.954, *P* = 0.046; *r* = −0.981, *P* = 0.019, respectively) (Supplementary Table 1).

**TABLE 3:** Soil alpha diversity in different land use types.

	Bacteria				Fungi			
Site	Chao1 richness	Shannon diversity	Simpson’s evenness	Good’s coverage (%)	Chao1 richness	Shannon diversity	Simpson’s evenness	Good’s coverage (%)
EL	3393.42 ± 250.14a	9.35 ± 0.05a	0.99 ± 0.01a	98.32 ± 0.06b	637.71 ± 137.96a	2.99 ± 1.73b	0.65 ± 0.31a	99.85 ± 0.04b
ML	2843.69 ± 28.87b	8.41 ± 0.22b	0.99 ± 0.00a	98.83 ± 0.19a	819.67 ± 42.05a	4.37 ± 0.10ab	0.88 ± 0.02a	99.82 ± 0.01a
GL	2776.62 ± 316.68b	8.73 ± 0.39b	0.99 ± 0.00a	98.87 ± 0.05a	785.36 ± 97.29a	4.31 ± 0.31ab	0.88 ± 0.03a	99.78 ± 0.01a
FL	3650.74 ± 164.91a	9.45 ± 0.20a	1.00 ± 0.00a	98.15 ± 0.12b	834.83 ± 149.59a	5.49 ± 1.00a	0.92 ± 0.07a	99.86 ± 0.05b

*EL, exclosure; ML, mowed land; GL, grazed land; and FL, farmland.*

*Values are the means ± SEs (n = 3).*

*Different letters represent significant differences between the means (P < 0.05).*

**FIGURE 3:**
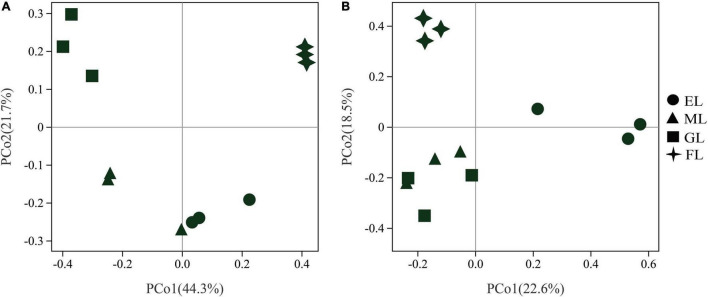
Principal component analysis (PCoA) of bacterial **(A)** and fungal **(B)** communities in different land use types. EL, exclosure; ML, mowed land; GL, grazed land; and FL, farmland.

Principal coordinates analysis was performed to visualize the changes in microbial community composition based on the Bray–Curtis distance (Figure 3). The first two axis of the PCoA explained 44.3 and 21.7% of the overall variation in the bacterial composition (Figure 3A). The PERMANOVA results showed that the profiles of the bacterial communities in the four land use types were clearly separated (*R*^2^ = 0.7099, *P* = 0.001), and BETADISPER was not significantly different (*P* = 0.08) (Supplementary Figure 3A). The bacterial communities in the GL and FL were significantly different from those in the EL and ML, and similar community compositions were observed in the EL and ML. For the fungi, the first two PCoA axis explained 22.6 and 18.5% of the overall variation in the community composition (Figure 3B). The PERMANOVA indicated that there was a significant difference in fungal community composition under different land use types (*R*^2^ = 0.4802, *P* = 0.001), and analyses of dispersion were no significant (*P* = 0.93) (Supplementary Figure 3B). Moreover, the fungal community in the EL clustered relatively far from those in the GL, FL, and ML along the PCo1 axis, which indicates that the structure of the soil fungal community in the EL differed markedly from those in the other land use types.

**FIGURE 4:**
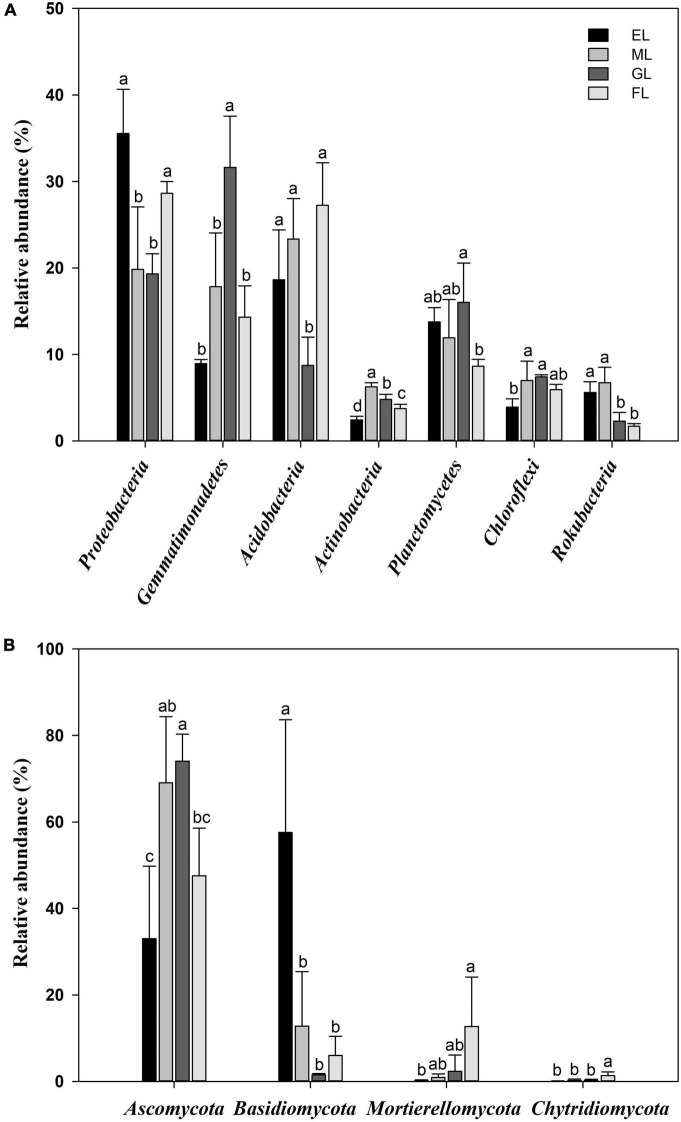
Relative abundances of the dominant bacterial taxa **(A)** and fungal taxa **(B)** at the phylum level in different land use types. Different letters indicate a significant difference (*P* < 0.05). EL, exclosure; ML, mowed land; GL, grazed land; and FL, farmland.

Considering all sequences, the dominant phyla of soil bacteria across all samples (with relative abundance >4%) were *Proteobacteria* (25.8%), *Acidobacteria* (19.5%), *Gemmatimonadetes* (18.2%), *Planctomycetes* (12.6%), *Chloroflexi* (6.1%), *Actinobacteria* (4.3%), and *Rokubacteria* (4.1%) in the soil profiles (Figure 4A and Supplementary Figure 1A). In detail, compared to the GL and ML, the EL and FL had a significantly higher relative abundance of *Proteobacteria* and a significantly lower relative abundance of *Chloroflexi* (no significant in FL). The GL had a higher abundance of *Gemmatimonadetes* and a lower abundance of *Acidobacteria* than the other land use types. The highest abundances of *Actinobacteria* and *Rokubacteria* were observed in the ML. Moreover, LEfSe analysis with a threshold of 2 showed that *Acidobacteria* was enriched in FL; *Proteobacteria* in EL; *Gemmatimonadetes* in GL; and *Actinobacteria* in ML (Supplementary Figure 2A). The dominant fungal phyla across all the plots were *Ascomycota* (55.9%), followed by *Basidiomycota* (19.5%), *Mortierellomycota* (6.5%), and *Chytridiomycota* (0.8%) (Figure 4B and Supplementary Figure 1B). The highest abundance of *Ascomycota* was found in the GL. Similarly, the relative abundance of *Basidiomycota* in the EL was considerably different from that in the other land use types. Significantly higher relative abundances of *Mortierellomycota* and *Chytridiomycota* were also recorded in the FL than in the other land use types. In addition, the LEfSe analysis revealed that *Ascomycota* was the fungal indicator group in the ML and GL; *Basidiomycota* in the EL; and *Ascomycota*, *Basidiomycota*, and *Chytridiomycota* in the FL (Supplementary Figure 2B). In addition, in the analyzed plots, the abundance of *Proteobacteria* was positively correlated with TN (*r* = 0.989, *P* = 0.011), while the abundance of *Ascomycota* was negatively correlated with TN (*r* = −0.972, *P* = 0.028) (Supplementary Tables 2, 3).

### Relationships Between Biotic and Abiotic Properties and Microbial Communities

**FIGURE 5:**
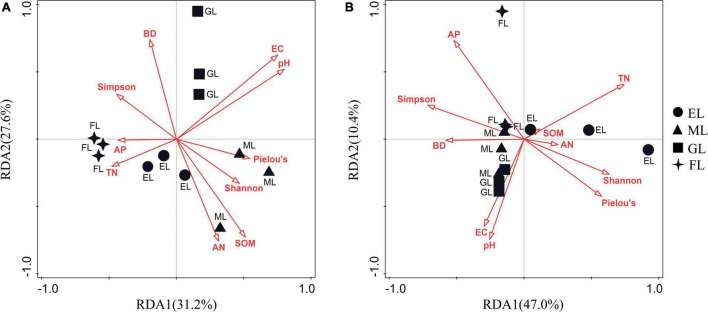
Redundancy analyses (RDA) of pooled data on bacterial **(A)** and fungal **(B)** communities with biotic and abiotic variables. The red vectors represent trajectories of environmental parameters as independent variables. EL, exclosure; ML, mowed land; GL, grazed land; and FL, farmland.

We performed RDA to illustrate the relationship between microbial community composition and environmental factors. The first two RDA axis explained 58.8 and 57.4% of the total variation in the overall compositions of the bacterial and fungal communities, respectively (Figure 5). The RDA results showed that EC (*P* = 0.002), SOM (*P* = 0.004), and Pielou’s evenness index for plants (*P* = 0.008) significantly influenced the composition of the bacterial community, while TN (*P* = 0.002) and the Shannon diversity of plants (*P* = 0.014) significantly affected the composition of the fungal community (Supplementary Table 4).

**FIGURE 6:**
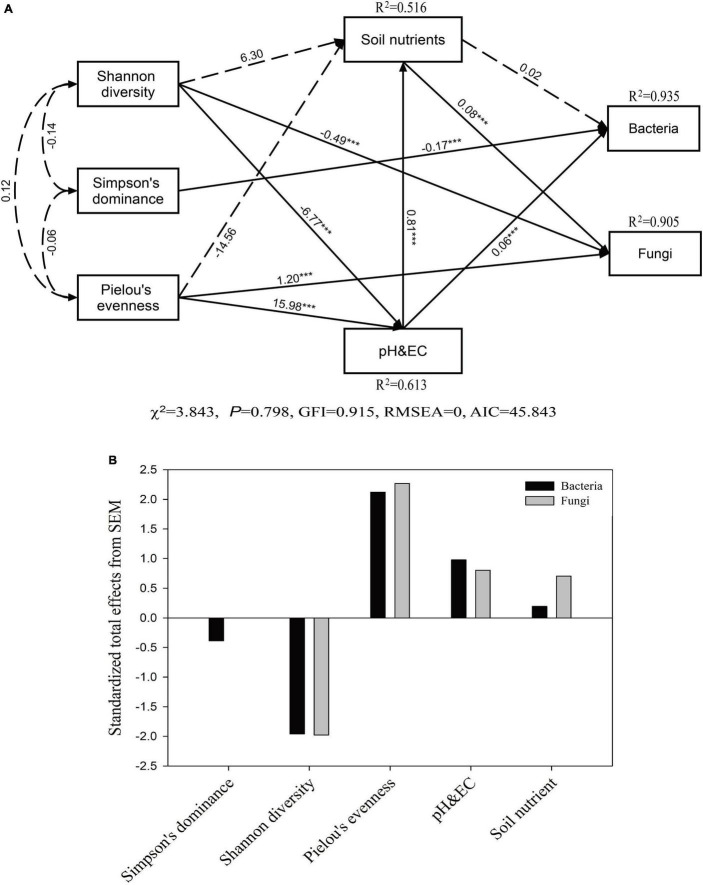
Structural equation model (SEM) based on the effects of plant community composition (Shannon, Simpson’s, and Pielou’s indexes) and soil properties (soil nutrients, pH, and EC) on soil microbial communities **(A)** and the standardized total effects on plant community composition and soil properties based on the SEM **(B)**. Continuous arrows represent positive or negative relationships, and adjacent numbers in the same direction as the arrows indicate path coefficients. The *R*^2^ values indicate the proportion of variance explained by each variable in the model. The significance levels are denoted as follow: ***P < 0.001.

The model provided a good fit among vegetation parameters, soil properties, and microbial community composition (*P* = 0.798, χ^2^ = 3.843, GFI = 0.915, RMSEA = 0.000, AIC = 45.843) (Figure 6). Our SEM results showed that soil pH and EC exhibited a direct positive correlation with the soil bacterial community (*P* < 0.001), while the plant Simpson index had a direct negative effect on the soil bacterial community (*P* < 0.001). Plant taxonomic diversity (Shannon diversity and Pielou’s evenness) affected the bacterial community composition indirectly by changing the soil pH and EC. The value of Pielou’s evenness index of plants directly and positively affected the soil pH and EC and fungal community composition (*P* < 0.001). In addition, the value of the Shannon diversity index of plants had a significantly negative correlation with soil pH and EC and the fungal community composition (*P* < 0.001), while the soil nutrient contents had a significantly positive and direct effect on the soil fungal community composition (*P* < 0.001).

## Discussion

### Effects of Land Use Types on Soil and Plant Properties

The biotic and abiotic properties represented strong changes that varied with land use type and were correlated with higher microbial activity, more plant litter, and more intense human disturbance (Bargali et al., 2019). The GL had a higher pH and EC due to the return of animal dung and urine to the soil surface. In contrast, our results showed that the FL had a lower soil pH and EC. On the one hand, the amount of fertilizer applied in agricultural production is often greater than the plant absorption capacity, leading to the loss of some fertilizer from the soil through leaching; this process is accompanied by the loss of many alkaline cations, while the H^+^ produced in the nitrification process remains in the soil, reducing the soil pH (Schirmel et al., 2018). On the other hand, crop harvesting removes biomass (e.g., grains, straw) and OH^–^ from plants, which aggravates the surplus of acid in the soil. Interestingly, we also noticed that a higher content of phosphorus was found in the FL than in the other land use types, which may be explained by the input of chemical fertilizer to offset the loss of nutrients. Many studies have shown that SOC and TN are important components of soil fertility and are closely related to ecosystem stability and environmental sustainability (Shi et al., 2020). Grazing consumed most of the aboveground plant biomass and promoted soil compaction, which significantly decreased plant diversity and richness, leading to a smaller quantity of plant litter inputs to the soil and thus lower C and N contents (Yang et al., 2019; Oggioni et al., 2020). The EL, which was not used for grazing or mowing for 3 years, had relatively high plant biomass and diversity (according to Shannon and Pielou’s indexes, respectively), which contributed to minimizing soil erosion and nutrient leaching; thus, the nutrients released by litter decomposition accumulated in the soil (Huhe et al., 2017). Appropriate annual mowing increased plant richness and diversity by reducing the competitive exclusion of dominant species and accelerated soil nutrient cycling (e.g., N and P cycling) (Wang et al., 2021). In addition, mowing increased the SOM content by increasing root exudates and plant residues during growth, resulting in a continuous flow of carbon into the soil (Chan et al., 2011).

### Effects of Land Use Types on Soil Microbial Community Diversity and Composition

Land use type had a strong driving effect on microbial community diversity. In this study, higher bacterial α diversity (measured by the Chao1 richness and Shannon indexes) occurred in the EL and FL. Undisturbed grasslands have rich plant communities, large amounts of biomass and litter, and diverse root exudates, which create a suitable habitat for soil microorganisms, leading to structural changes in the soil bacterial community and a positive relationship between plants and microbial diversity (Zhang et al., 2016). After the grassland was converted to farmland, frequent disturbances caused a decrease in abundances of the dominant OTUs and an increase in diversity, and OTUs were replaced by competitors (George et al., 2019). Grazing or mowing resulted in lower returns of organic carbon, nitrogen, and other nutrients to the soil and reduced available substrate resources for microorganisms, resulting in intensified competition. All these factors affect the soil environment and reduce the soil bacterial diversity (Oggioni et al., 2020). In addition, our study also detected a significantly higher Shannon index among the fungi in the FL across all plots. Human activities, especially agricultural management, have an evident effect on the composition of vegetation, the levels of soil water and heat, and the mineralization of soil organic matter, leading to structural changes in the soil fungal community that can lead to the emergence of diversity and new species (Arévalo-Gardini et al., 2020). Fungi can adapt to phosphorus input levels, giving them a competitive advantage over many bacteria (Yan and Feng, 2020). Therefore, increasing soil nutrients through fertilizer application might also have a positive effect on fungal communities.

Land use change also affected the microbial community composition and reshaped the unique keystone taxa in our study. Among the bacteria, the rate of species replacement in the GL was relatively fast, which might be due to soil nutrient scarcity and intensified competition, leading to intensified species replacement among communities. The abundance of the phylum *Gemmatimonadetes* was higher in the GL than in the other land use types; members of this phylum contribute to the recycling of essential nutrients and decomposition of cellulose and lignin and are positively correlated with soil bulk density (Xu et al., 2019). In contrast, fertilization results in a large input of nutrients to enhance the metabolic activity of *Acidobacteria*, which is important for soil carbon and nitrogen cycling (Kielak et al., 2016). The high *Proteobacteria* abundance in the EL was consistent with the results reported by Ding et al. (2017), who observed a relatively high abundance of *Proteobacteria* in soils with high nitrogen contents. Moreover, the abundance of *Actinobacteria*, which contributed to the degradation and mineralization of litter in the soil and may have contributed to the high SOC contents in the plots, was higher in the ML than in the other land use types (Yu et al., 2020). In addition, the bacterial communities in the EL and ML were more similar than those in the other land use types, indicating that the EL and ML had more similar substrate compositions. The accumulation of recalcitrant organic compounds in the EL supports the activities of fungi associated with the decomposition of recalcitrant compounds, i.e., an increase in the number of *Basidiomycota*, which triggered a change in the soil fungal community structure (Wang J. C. et al., 2020). In addition, *Chytridiomycota* were recovered in the FL, probably due to their ability to tolerate regular use of pesticides and fertilizers, which are continuously required in agricultural practices (Gleason et al., 2004). Similarly, the abundance of *Mortierellomycota*, which have utilized simple carbohydrates efficiently, was higher in the FL than in the other land use types.

### Relationships Between Biotic and Abiotic Properties and Microbial Communities

Land use change had an obvious impact on the soil bacterial and fungal community compositions in the Songnen grassland ecosystem. Studies have found that soil pH is equally important as salinity in shaping bacterial communities in saline soils (Zhao et al., 2018). Generally, soil pH has long been recognized as a primary driver for bacterial communities (Tripathi et al., 2018). On the other hand, high soil salinity (EC) could limit plant water and nutrient uptake due to low osmotic potential and negatively affect the biomass, activity, and biochemical processes of soil microorganisms (Yang et al., 2020). Our structural model confirms this finding. In our study, soil pH and EC exhibited collinearity and were highly correlated with bacterial diversity, which affected the composition of the bacterial community. Notably, plant diversity may directly affect the soil microbial community structure through resource allocation and nutrient supply, and can also regulate bacterial communities by changing the soil environment (Shen et al., 2021). In this study, frequent grazing or mowing removed a large number of species from the plant community, thus reducing plant diversity and affecting bacterial distribution indirectly by changing soil pH and EC. In addition, the SEM provided evidence that plant and soil properties were highly correlated with the fungal community structure. Not surprisingly, changes in soil nutrients directly affect fungal communities (Huhe et al., 2017). We further found that plant diversity seemed to play a key role in changes in the fungal communities. Plant richness could drive the distribution of soil microorganisms, which may be explained by the fact that higher species richness will modify resource availability and microclimates, creating more niches in the soil and altering the soil fungal composition (Chen et al., 2018a). On the other hand, communities with increased plant richness have a higher chance of including key plant species, and these key species or functional groups will strongly influence the soil fungal composition (Shen et al., 2021). In addition, our study also found that the Shannon diversity of the plant communities had a negative effect on the fungal composition. A study by Chen et al. (2018b) found that soil microbes depend more on the presence of a specific plant than on plant diversity itself.

It is worth mentioning that pseudo-replication is a limitation of this study. The effects of land use types on bacterial and fungal communities were studied within a region rather than across multiple regions, so our different sampling sites should be regarded as geographical pseudo-replications. This limitation should be addressed in future studies. However, since each site within a given region was separated by at least 2 km, we believe that the site effects are almost certainly than any possible random differences, and inferences are drawn from pseudo-replicated designs.

## Conclusion

In summary, this study revealed the relationship between the microbial community composition and plant and soil properties in different grassland uses of the Songnen Plain. Our study found that changes in land use types distinctly influenced on the plant community composition, soil properties, and abundances of dominant microbial groups. In addition, we found that plant community evenness, as measured by Pielou’s evenness index and soil nutrient contents might be the key drivers of fungal community formation, whereas the bacterial community was more affected by changes in pH and EC (salinity) during land use. Further research should focus on the links between soil microbial communities and functions to address how land use types affect important ecosystem processes.

## Data Availability Statement

The high-throughput sequencing data have been deposited in the Sequence Read Archive (SRA) of the NCBI database under the BioProject ID PRJNA805040: https://www.ncbi.nlm.nih.gov/sra/PRJNA805040.

## Author Contributions

GL, ZB, and GC conceived and designed the study. GL drafted the original manuscript. ZB provided constructive suggestions for revisions. WH provided to the study site. ZK, GJ, HG, and DL contributed to the sampling and data analysis. All authors read and approved the final manuscript.

## Conflict of Interest

The authors declare that the research was conducted in the absence of any commercial or financial relationships that could be construed as a potential conflict of interest.

## Publisher’s Note

All claims expressed in this article are solely those of the authors and do not necessarily represent those of their affiliated organizations, or those of the publisher, the editors and the reviewers. Any product that may be evaluated in this article, or claim that may be made by its manufacturer, is not guaranteed or endorsed by the publisher.
