# Supplementary material for: Effects of Land Use on the Soil Microbial Community in the Songnen Grassland of Northeast China
Source: Front Microbiol. 2022 Jul 8;13:865184. doi: 10.3389/fmicb.2022.865184 (PMC9307977; doi:10.3389/fmicb.2022.865184)
Supplement: Supplementary file 1 [file Data_Sheet_1.docx]

**Supporting Information**

**Supplementary Figures and Tables**


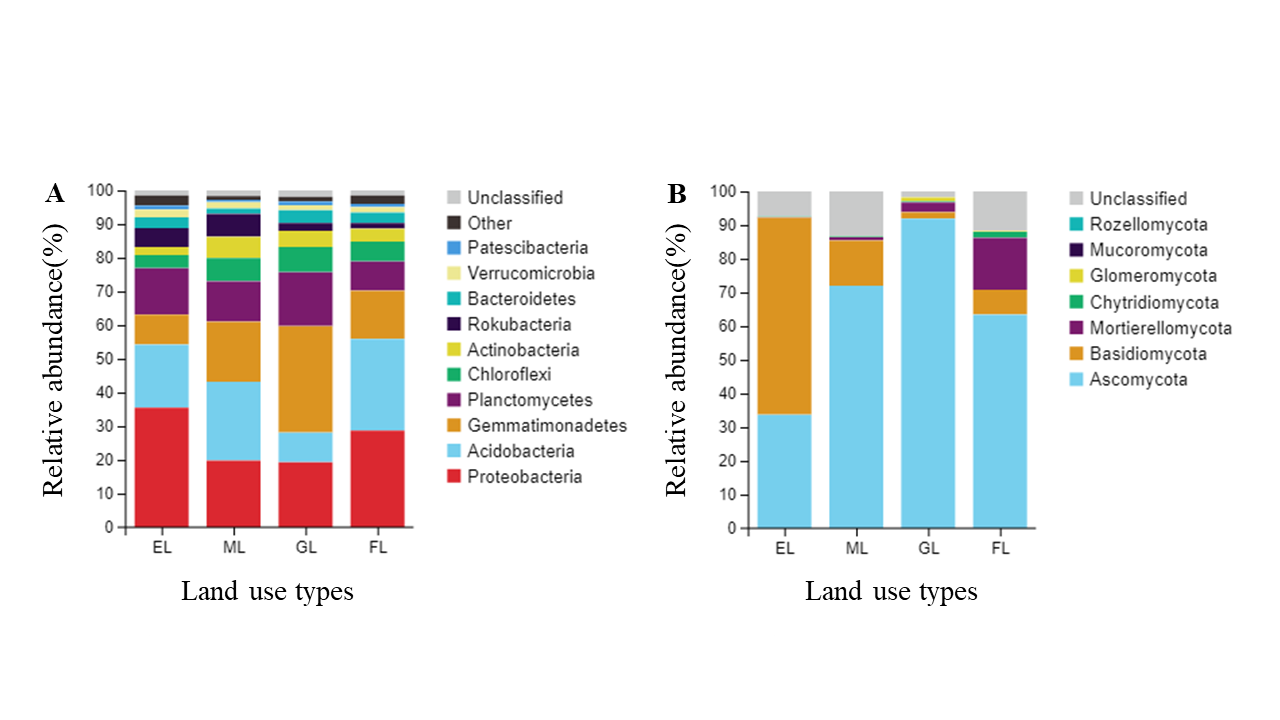


**land use types**

**FIGURE S1 |** Relative abundances of the total bacterial phyla (**A**) and total fungal phyla (**B**) in different land use types. EL (exclosure), ML (mowed land), GL (grazed land), and FL (farmland).


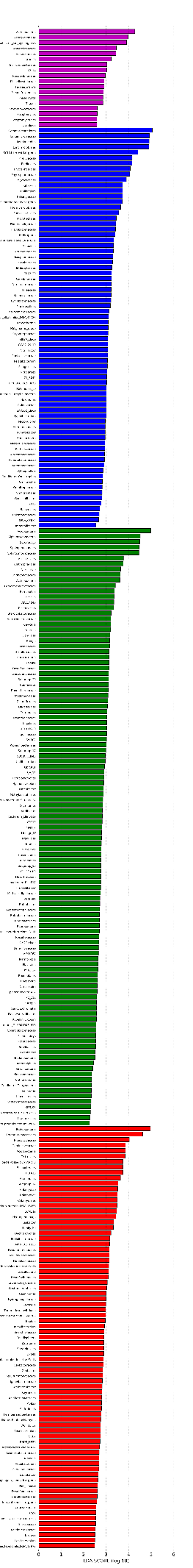


**A**


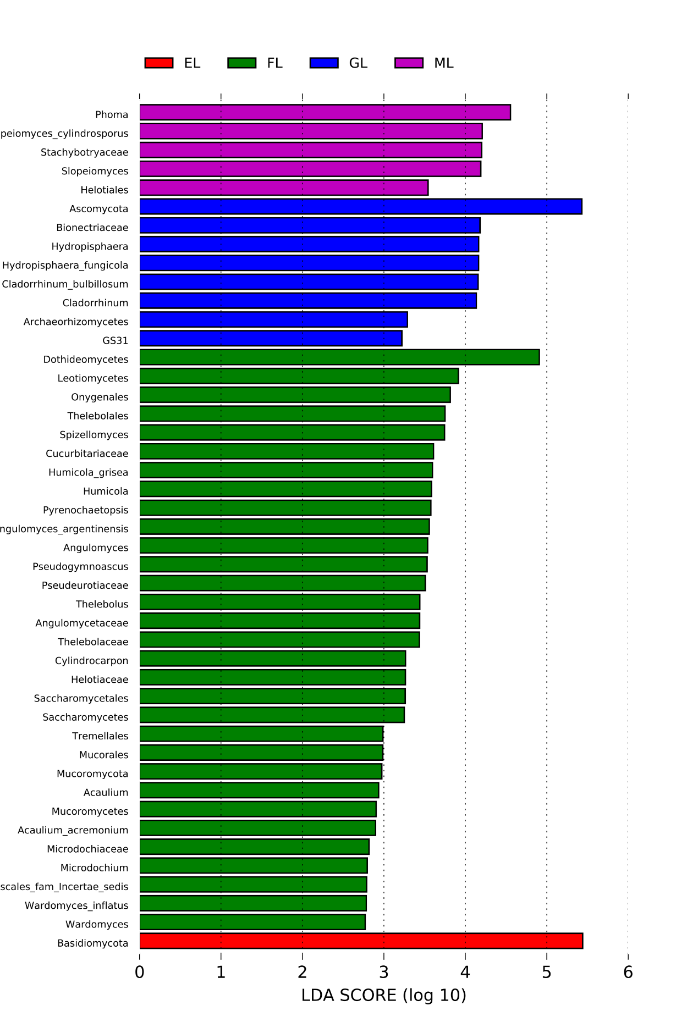


**B**

**FIGURE S2 |** Indicator bacteria with linear discriminant analysis (LDA) scores of 2 or greater in the bacterial communities (**A**), and indicator fungi with LDA scores of 2 or greater in the fungal communities (**B**) in different land use types. The different colors represent different treatments and dominant phyla. EL (exclosure), ML (mowed land), GL (grazed land), and FL (farmland).


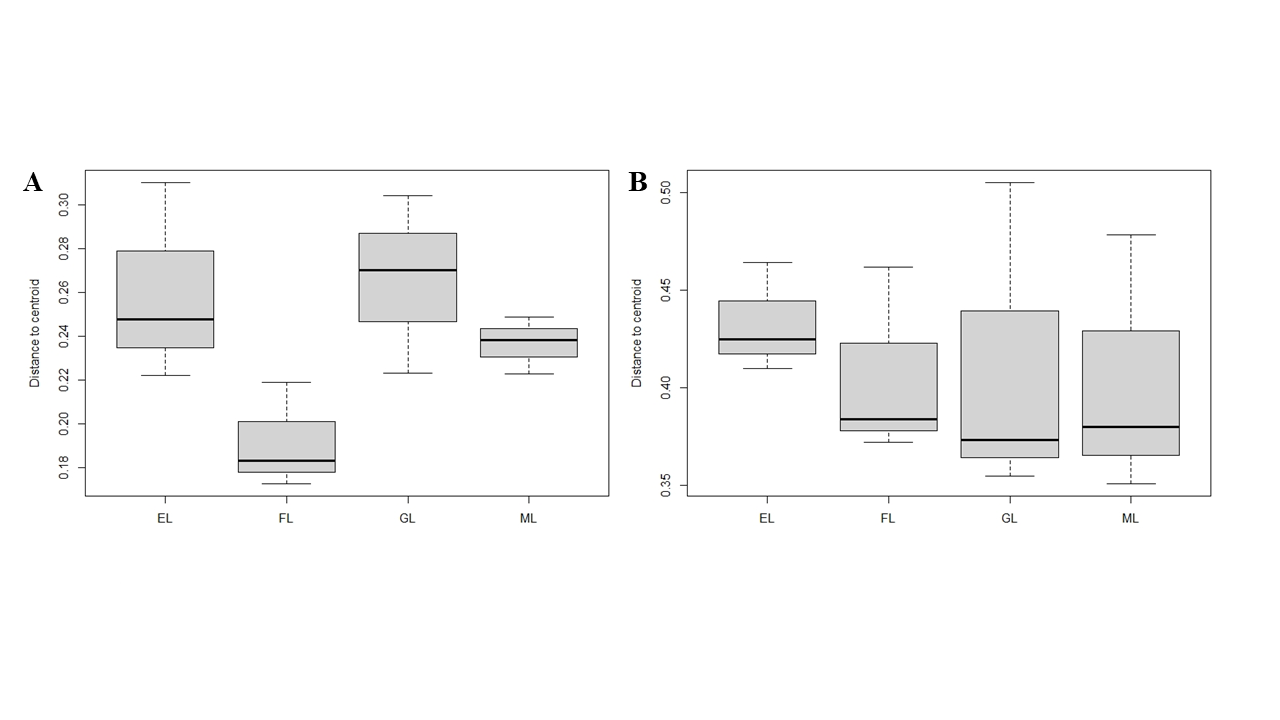


**FIGURE S3 |** Dispersion of the composition of bacterial (**A**) and fungal (**B**) OTUs (expressed as the distance to the centroids of each site). Results of dispersion of variances of groups (Permutation test: *F*_bacteria_ = 3.05, *P* = 0.08; *F*_fungi_ = 0.15, *P* = 0.93) were no significant. EL (exclosure), ML (mowed land), GL (grazed land), and FL (farmland).

**TABLE S1 |** Spearman's rank correlations between alpha-diversities and environmental factors.

| **Variable** | | **pH** | **EC** | **SOM** | **TN** | **BD** | **AP** | **AN** | **Plant** | | | **Bacteria** | | | **Fungi** | | |
| --- | --- | --- | --- | --- | --- | --- | --- | --- | --- | --- | --- | --- | --- | --- | --- | --- | --- |
|  |  |  |  |  |  |  |  |  | **Shannon** | **Simpson** | **Pielou's** | **BShannon** | **BSimpson** | **BChao1** | **FShannon** | **FSimpson** | **FChao1** |
| pH | | 1 |  |  |  |  |  |  |  |  |  |  |  |  |  |  |  |
| EC | | 0.992^**^ | 1 |  |  |  |  |  |  |  |  |  |  |  |  |  |  |
| SOM | | -0.057 | -0.129 | 1 |  |  |  |  |  |  |  |  |  |  |  |  |  |
| TN | | -0.638 | -0.709 | -0.014 | 1 |  |  |  |  |  |  |  |  |  |  |  |  |
| BD | | 0.302 | 0.414 | -0.788 | -0.584 | 1 |  |  |  |  |  |  |  |  |  |  |  |
| AP | | -0.443 | -0.332 | -0.229 | -0.364 | 0.544 | 1 |  |  |  |  |  |  |  |  |  |  |
| AN | | -0.212 | -0.298 | 0.972* | 0.233 | -0.905 | -0.304 | 1 |  |  |  |  |  |  |  |  |  |
| Plant | Shannon | 0.165 | 0.039 | 0.544 | 0.487 | -0.838 | -0.912 | 0.651 | 1 |  |  |  |  |  |  |  |  |
|  | Simpson | 0.996** | 0.980* | 0.027 | -0.624 | 0.224 | -0.482 | -0.126 | 0.229 | 1 |  |  |  |  |  |  |  |
|  | Pielou's | 0.365 | 0.246 | 0.397 | 0.385 | -0.678 | -0.983* | 0.472 | 0.969^*^ | 0.417 | 1 |  |  |  |  |  |  |
| Bacteria | Shannon | -0.812 | -0.807 | -0.425 | 0.805 | -0.073 | 0.186 | -0.221 | -0.124 | -0.841 | -0.209 | 1 |  |  |  |  |  |
|  | Simpson | -0.758 | -0.670 | -0.348 | 0.103 | 0.365 | 0.884 | -0.309 | -0.768 | -0.799 | -0.874 | 0.622 | 1 |  |  |  |  |
|  | Chao1 | -0.967^*^ | -0.955^*^ | -0.172 | 0.719 | -0.183 | 0.384 | 0.007 | -0.192 | -0.979^*^ | -0.350 | 0.933 | 0.760 | 1 |  |  |  |
| Fungi | Shannon | -0.244 | -0.125 | -0.281 | -0.539 | 0.671 | 0.977* | -0.396 | -0.954^*^ | -0.288 | -0.981^*^ | 0.021 | 0.782 | 0.194 | 1 |  |  |
|  | Simpson | 0.145 | 0.259 | -0.209 | -0.827 | 0.741 | 0.819 | -0.396 | -0.873 | 0.107 | -0.837 | -0.355 | 0.474 | -0.209 | 0.919 | 1 |  |
|  | Chao1 | 0.078 | 0.185 | -0.066 | -0.811 | 0.628 | 0.836 | -0.253 | -0.827 | 0.051 | -0.825 | -0.375 | 0.484 | -0.180 | 0.918 | 0.988^*^ | 1 |

AN (alkali-hydrolyzable nitrogen), AP (available phosphorus), SOM (soil organic matter), EC (electrical conductivity), TN (total nitrogen), BD (bulk density).

**P* < 0.05; ***P* < 0.01.

**TABLE S2 |** Spearman's rank correlations between the relative abundances of the dominant bacterial phyla and environmental factors.

| **Variable** | **pH** | **EC** | **SOM** | **TN** | **BD** | **AP** | **AN** | **Plant Shannon** | **Plant**  **Simpson** | **Plant Pielou's** | **Pro** | **Gem** | **Aci** | **Act** | **Pla** | **Chl** | **Rok** | **Ver** | **Bac** |
| --- | --- | --- | --- | --- | --- | --- | --- | --- | --- | --- | --- | --- | --- | --- | --- | --- | --- | --- | --- |
| pH | 1 |  |  |  |  |  |  |  |  |  |  |  |  |  |  |  |  |  |  |
| EC | 0.992^**^ | 1 |  |  |  |  |  |  |  |  |  |  |  |  |  |  |  |  |  |
| SOM | -0.057 | -0.129 | 1 |  |  |  |  |  |  |  |  |  |  |  |  |  |  |  |  |
| TN | -0.638 | -0.709 | -0.014 | 1 |  |  |  |  |  |  |  |  |  |  |  |  |  |  |  |
| BD | 0.302 | 0.414 | -0.788 | -0.584 | 1 |  |  |  |  |  |  |  |  |  |  |  |  |  |  |
| AP | -0.443 | -0.332 | -0.229 | -0.364 | 0.544 | 1 |  |  |  |  |  |  |  |  |  |  |  |  |  |
| AN | -0.212 | -0.298 | 0.972* | 0.233 | -0.905 | -0.304 | 1 |  |  |  |  |  |  |  |  |  |  |  |  |
| Plant Shannon | 0.165 | 0.039 | 0.544 | 0.487 | -0.838 | -0.912 | 0.651 | 1 |  |  |  |  |  |  |  |  |  |  |  |
| Plant  Simpson | 0.996** | 0.980* | 0.027 | -0.624 | 0.224 | -0.482 | -0.126 | 0.229 | 1 |  |  |  |  |  |  |  |  |  |  |
| Plant  Pielou's | 0.365 | 0.246 | 0.397 | 0.385 | -0.678 | -0.983* | 0.472 | 0.969^*^ | 0.417 | 1 |  |  |  |  |  |  |  |  |  |
| Pro | -0.747 | -0.806 | -0.009 | 0.989^*^ | -0.557 | -0.226 | 0.226 | 0.384 | 0.760 | 0.257 | 1 |  |  |  |  |  |  |  |  |
| Gem | 0.823 | 0.885 | -0.491 | -0.774 | 0.789 | 0.052 | -0.664 | -0.400 | -0.734 | -0.179 | -0.826 | 1 |  |  |  |  |  |  |  |
| Aci | -0.811 | -0.796 | 0.482 | 0.196 | -0.363 | 0.584 | 0.522 | -0.203 | 0.775 | -0.438 | 0.325 | -0.724 | 1 |  |  |  |  |  |  |
| Act | 0.581 | 0.620 | 0.377 | -0.931 | 0.252 | 0.242 | 0.147 | -0.240 | -0.780 | -0.200 | -0.921 | 0.544 | -0.018 | 1 |  |  |  |  |  |
| Pla | 0.825 | 0.774 | -0.211 | -0.103 | 0.074 | -0.788 | -0.237 | 0.481 | 0.598 | 0.682 | -0.249 | 0.562 | -0.952* | 0.032 | 1 |  |  |  |  |
| Chl | 0.642 | 0.723 | -0.134 | -0.989* | 0.695 | 0.393 | -0.365 | -0.564 | 0.820 | -0.438 | -0.979* | 0.841 | -0.267 | 0.867 | 0.135 | 1 |  |  |  |
| Rok | 0.141 | 0.038 | 0.923 | 0.119 | -0.863 | -0.585 | 0.926 | 0.819 | 0.615 | 0.718 | 0.067 | -0.416 | 0.163 | 0.231 | 0.142 | -0.254 | 1 |  |  |
| Ver | -0.263 | -0.383 | 0.572 | 0.745 | -0.951* | -0.702 | 0.732 | 0.908 | 0.225 | 0.792 | 0.690 | -0.742 | 0.150 | -0.477 | 0.117 | -0.822 | 0.745 | 1 |  |
| Bac | 0.044 | 0.082 | -0.955^*^ | 0.247 | 0.584 | -0.031 | -0.874 | -0.292 | -0.198 | -0.137 | 0.213 | 0.356 | -0.567 | -0.574 | 0.353 | -0.103 | -0.785 | -0.311 | 1 |

AN (alkali-hydrolyzable nitrogen), AP (available phosphorus), SOM (soil organic matter), EC (electrical conductivity), TN (total nitrogen), BD (bulk density).

The bacterial communities included Pro (*Proteobacteria*), Gem (*Gemmatimonadetes*), Aci (*Acidobacteria*), Act (*Actinobacteria*), Pla (*Planctomycetes*), Chl (*Chloroflexi*), Rok (*Rokubacteria*), Ver (*Verrucomicrobia*), and Bac (*Bacteroidetes*).

**P* < 0.05; ***P* < 0.01.

**TABLE S3 |** Spearman's rank correlations between the relative abundances of the dominant fungal phyla and environmental factors.

| **Variable** | **pH** | **EC** | **SOM** | **TN** | **BD** | **AP** | **AN** | **Plant Shannon** | **Plant Simpson** | **Plant Pielou's** | **Asc** | **Bas** | **Mor** | **Chy** |
| --- | --- | --- | --- | --- | --- | --- | --- | --- | --- | --- | --- | --- | --- | --- |
| pH | 1 |  |  |  |  |  |  |  |  |  |  |  |  |  |
| EC | 0.992^**^ | 1 |  |  |  |  |  |  |  |  |  |  |  |  |
| SOM | -0.057 | -0.129 | 1 |  |  |  |  |  |  |  |  |  |  |  |
| TN | -0.638 | -0.709 | -0.014 | 1 |  |  |  |  |  |  |  |  |  |  |
| BD | 0.302 | 0.414 | -0.788 | -0.584 | 1 |  |  |  |  |  |  |  |  |  |
| AP | -0.443 | -0.332 | -0.229 | -0.364 | 0.544 | 1 |  |  |  |  |  |  |  |  |
| AN | -0.212 | -0.298 | 0.972* | 0.233 | -0.905 | -0.304 | 1 |  |  |  |  |  |  |  |
| Plant Shannon | 0.165 | 0.039 | 0.544 | 0.487 | -0.838 | -0.912 | 0.651 | 1 |  |  |  |  |  |  |
| Plant Simpson | 0.996** | 0.980* | 0.027 | -0.624 | 0.224 | -0.482 | -0.126 | 0.229 | 1 |  |  |  |  |  |
| Plant Pielou's | 0.365 | 0.246 | 0.397 | 0.385 | -0.678 | -0.983* | 0.472 | 0.969^*^ | 0.417 | 1 |  |  |  |  |
| Asc | 0.799 | 0.855 | -0.046 | -0.972* | 0.579 | 0.163 | -0.277 | -0.355 | 0.783 | -0.208 | 1 |  |  |  |
| Bas | -0.319 | -0.432 | 0.334 | 0.874 | -0.841 | -0.707 | 0.530 | 0.848 | -0.272 | 0.758 | -0.793 | 1 |  |  |
| Mor | -0.652 | -0.551 | -0.441 | -0.011 | 0.501 | 0.916 | -0.426 | -0.853 | -0.703 | -0.927 | -0.167 | -0.468 | 1 |  |
| Chy | -0.689 | -0.592 | -0.358 | -0.006 | 0.438 | 0.927 | -0.344 | -0.828 | -0.733 | -0.922 | -0.186 | -0.447 | 0.996* | 1 |

AN (alkali-hydrolyzable nitrogen), AP (available phosphorus), SOM (soil organic matter), EC (electrical conductivity), TN (total nitrogen), BD (bulk density).

The fungal communities included Asc (*Ascomycota*), Bas (*Basidiomycota*), Mor (*Mortierellomycota*), and Chy (*Chytridiomycota*).

**P* < 0.05; ***P* < 0.01.

**TABLE S4 |** Correlations of the soil bacterial community composition with soil and plant parameters.

| **Variable** | **Bacteria** | | |
| --- | --- | --- | --- |
|  | **Explains（%）** | ***F*** | ***P*** |
| EC | 28.6 | 4.0 | **0.002** |
| SOM | 24.2 | 4.6 | **0.004** |
| Plant Pielou’s | 9.6 | 2.0 | **0.008** |
| TN | 6.9 | 1.5 | 0.152 |
| BD | 6.2 | 1.4 | 0.206 |
| Plant Shannon | 4.6 | 1.1 | 0.378 |
| pH | 4.4 | 0.7 | 0.588 |
| Plant Simpson | 3.7 | 0.9 | 0.550 |
| AP | 3.3 | 0.7 | 0.588 |
| AN | 3.0 | 0.6 | 0.666 |

Alkali-hydrolyzable nitrogen (AN), available phosphorus (AP), bulk density (BD), total nitrogen (TN), Electrical conductivity (EC), soil organic matter (SOM).

**TABLE S5 |** Correlations of the soil fungal community composition with soil and plant parameters.

| **Variable** | **Fungi** | | |
| --- | --- | --- | --- |
|  | **Explains（%）** | ***F*** | ***P*** |
| TN | 28.8 | 4.0 | **0.002** |
| Plant Shannon | 14.6 | 2.9 | **0.014** |
| AP | 14.4 | 2.3 | 0.066 |
| EC | 6.9 | 1.5 | 0.206 |
| Plant Simpson | 6.9 | 1.1 | 0.366 |
| Plant Pielou’s | 6.3 | 1.4 | 0.206 |
| BD | 5.1 | 1.2 | 0.324 |
| pH | 4.4 | 0.8 | 0.542 |
| AN | 3.7 | 0.8 | 0.556 |
| SOM | 3.3 | 0.7 | 0.590 |

Alkali-hydrolyzable nitrogen (AN), available phosphorus (AP), bulk density (BD), total nitrogen (TN), Electrical conductivity (EC), soil organic matter (SOM).
